# Ophthalmology training and competency levels in caring for patients with ophthalmic complaints among United States internal medicine, emergency medicine, and family medicine residents

**DOI:** 10.3352/jeehp.2019.16.25

**Published:** 2019-08-29

**Authors:** Christopher Daniel Gelston, Jennifer Landrigan Patnaik

**Affiliations:** Department of Ophthalmology, University of Colorado School of Medicine, Aurora, CO, USA; Hallym University, Korea

**Keywords:** Accreditation, Cross-sectional studies, Graduate medical education, Internship and residency, Ophthalmology, United Sates

## Abstract

**Purpose:**

To evaluate ophthalmic educational training and confidence in caring for patients with ophthalmic complaints among internal, emergency, and family medicine residents in the United States.

**Methods:**

A 41-item cross-sectional survey was sent to the directors of 529 internal medicine, 237 emergency medicine, and 629 family medicine residency programs, who distributed it to residents in those programs. The survey included the number of ophthalmic education hours residents received. Respondents were asked to rate their confidence in performing an ophthalmic exam and treating patients with ocular conditions using a 5-point Likert scale ranging from “not confident” to “very confident.”

**Results:**

In total, 92.5% of internal medicine, 66.8% of emergency medicine, and 74.5% of family medicine residents received less than 10 hours of ophthalmic education during residency. Most respondents (internal medicine, 59.1%; emergency medicine, 76.0%; family medicine, 65.7%) reported that patients with ocular complaints constituted 1%–5% of visits. Mean±standard deviation confidence levels in performing an eye exam and treating patients with ophthalmic conditions were highest in emergency medicine residency programs (2.9±0.7), followed by family medicine (2.3±0.6) and internal medicine (2.2±0.6). A higher reported number of ophthalmic education hours in residency was associated with greater confidence among emergency (P<0.001), family (P<0.001), and internal (P=0.005) medicine residents.

**Conclusion:**

Internal, emergency, and family medicine residents receive limited ophthalmic education, as reflected by their overall low confidence levels in performing an ophthalmic exam and treating patients with ocular complaints. An increase in ophthalmic educational hours during their residencies is recommended to improve upon this knowledge gap.

## Introduction

Internal, emergency, and family medicine physicians are often the gateway to ophthalmic subspecialty care and should therefore have the necessary education to properly evaluate and treat patients with ocular complaints. A survey showed that only 18% of United States medical schools required ophthalmology education or clerkships [1]. One reason for this low percentage is the belief that ophthalmic education will be given during primary care residencies [2]. However, past evidence has shown that 85% of family medicine and internal medicine residency directors believe that a major component of ophthalmic education should occur during medical school [3]. Furthermore, a national survey of internal medicine clerkship directors reported that “core” physical exam components should include pupillary reaction to light, while “not core” physical exam skills included a fundoscopic exam and testing visual acuity [4].

Patients present to internal, emergency, and family medicine physicians with various ocular complaints, including red eye, eye pain, and decreased vision [5]. Studies have demonstrated that 3.4% of emergency department visits were for urgent and non-urgent ocular conditions [6] and that accurate diagnosis of eye conditions was achieved in between 35.9% (by primary healthcare providers) and 48.2% of cases (by emergency department providers) [7].

We studied internal medicine, emergency medicine, and family medicine residency programs in the United States to determine whether the amount of ophthalmology training residents receive was correlated with their confidence in examining, diagnosing, and treating patients with ophthalmic complaints.

## Methods

### Ethical statement

This study was approved by the Institutional Review Board at the University of Colorado in Aurora, CO, USA (18-1180). Informed consent was obtained from participating residents.

### Study design and participants

A 41-item cross-sectional survey questionnaire was electronically sent to residency program directors of 529 internal medicine, 237 emergency medicine, and 639 family medicine Accreditation Council for Graduate Medical Education (ACGME)-accredited programs in the United States during October 2018 (Appendix 1). A cover letter was included in the email with a link to REDCap (https://www.project-redcap.org/), and the recipients were asked to forward the anonymous survey to the first- (postgraduate year [PGY]-1), second- (PGY-2), third- (PGY-3), and fourth-year (PGY-4) residents in their respective programs. REDCap is a secure web-based application that has the capability to survey participants online. To increase participation in the study, a reminder email was sent 2 weeks after the initial communication.

### Survey description

The survey was designed to collect demographic data and to rate participants’ comfort levels in performing key components of an ophthalmic exam and treating patients with common and emergent ocular conditions. The initial part of the survey (9 items) inquired about demographics, hours of ophthalmology training in medical school, their respective residency program, and percentage of patients presenting with ocular complaints. The next 8 items asked the residents to rate their comfort levels in performing various aspects of the ophthalmic exam, including measuring visual acuity and assessing pupils. The remaining 24 items asked the residents to assess their confidence levels in treating and managing care for various ophthalmic diseases, including conjunctivitis, corneal abrasion, and chalazion. Survey questions regarding comfort levels and confidence levels used a 5-point Likert scale with the following options: 1, not confident; 2, mildly confident; 3, moderately confident; 4, confident; and 5, very confident. The validity of the survey was determined after survey administration by measuring the association between reported hours in residency dedicated to ophthalmology and the composite scores of respondents’ confidence levels. The reliability of the survey was measured by the standardized Cronbach α value of all the Likert-scale items.

### Statistical analysis

Data were collected directly into REDCap and downloaded into SAS ver. 9.4 (SAS Institute Inc., Cary, NC, USA) for statistical analysis. Measures for ophthalmic conditions, procedures/tests, and practices were self-reported with categorical answers and Likert-scale items. Each categorical variable was summarized with basic frequencies. The chi-square test was used to assess the different residency program types and hours of ophthalmic education. Likert-scale items were summarized by averages with associated standard deviations (SDs) to determine areas where training and comfort levels were highest and lowest. Comparisons of Likert-scale items across the 3 residency programs (internal medicine, emergency medicine, and family medicine) were made using analysis of variance (ANOVA). The composite scores of all Likert-scale items were created by averaging the 32 items that assessed confidence levels in performing various ophthalmology skills. ANOVA was also used to compare composite scores by hours dedicated to ophthalmic education and current residency year for each type of residency program.

## Results

Internal, emergency, and family medicine residents completed a total of 1,025 surveys across the United States. The respondents comprised 412 (40.2%) internal medicine residents, 342 (33.4%) emergency medicine residents, and 271 (26.4%) family medicine residents. The distribution of year of training was as follows: PGY-1, 376 (36.7%); PGY-2, 320 (31.2%); PGY-3, 291 (28.4%); and PGY-4, 37 (3.6%) (Table 1).

During residency, most respondents reported receiving less than 10 hours dedicated to ophthalmology during their training (internal medicine, 380 [92.5%]; family medicine, 202 [74.5%]; and emergency medicine, 227 [66.8%]). The majority of respondents in internal medicine (243 [59.1%]), emergency medicine (259 [76.0%]), and family medicine (178 [65.7%]) residencies reported that patients with ocular complaints constituted 1%–5% of visits (Table 2).

Confidence levels in performing an ophthalmic evaluation, managing care, and indications for prescribing ophthalmic drops among the different specialties are shown in Table 3. The standardized Cronbach α value for the Likert-scale questions was 0.955, indicating high reliability. Across the specialties, mean± SD scores indicated that residents felt “confident” assessing extraocular movements (4.1±0.9) and pupils (3.8±1.0), “moderately confident” in testing visual acuity (3.3±1.2), and “not confident to mildly confident” with slit lamp examinations (1.7±1.0). In managing ophthalmic disease, residents across the specialties felt “moderately confident” in treating conjunctivitis (3.5±1.0) and subconjunctival hemorrhage (3.1±1.3) and “mildly confident” in treating sight-threatening conditions, including corneal ulcer (2.2±1.2) and acute angle closure glaucoma (2.0±1.1). Systemic conditions often associated with ocular complaints, including thyroid eye disease (1.7±1.0), rheumatological conditions (1.6±0.9), and Sjögren syndrome (1.8±0.9) had the lowest reported mean confidence levels.

The subgroup analysis presented in Table 3 showed the highest composite score confidence levels in emergency medicine residents (2.9±0.7) and the lowest in internal medicine residents (2.2±0.7) and in family medicine residents (2.3±0.6). For sight-threatening emergencies, including corneal ulcer (3.2±1.2) and angle closure glaucoma (2.8±1.2), confidence levels were also noticeably higher in emergency medicine residents. Family medicine residents felt less confident in treating patients with a corneal ulcer (1.8±1.0) and angle closure glaucoma (1.7±0.9), as did internal medicine residents (1.7±0.9 and 1.6±0.9, respectively).

Table 4 demonstrates that higher numbers of ophthalmic education hours received by the residents in their emergency, internal, or family medicine residency programs were associated with an overall increase in their average confidence levels in emergency medicine programs (P<0.001) and family medicine programs (P<0.001), but not in internal medicine programs (P=0.01). In addition, the average confidence levels increased from PGY-1 to PGY-4 in emergency medicine programs (P<0.001), but not in internal medicine programs (P=0.05) or family medicine programs (P= 0.01). The raw data are available in Supplement 1.

## Discussion

Our results from this nationwide survey indicate a knowledge gap in terms of ophthalmic educational hours and confidence levels in treating patients with ocular complaints in emergency, internal, and family medicine residency programs. Three-quarters of all respondents across the specialties received less than 10 hours of ophthalmic education during their residency training program. There was an association between a higher number of ophthalmic education hours received by the residents from PGY-1 to PGY-4 and confidence levels in treating patients with ocular conditions. However, the mean composite scores in emergency medicine residents (2.9±0.7) were only considered “moderately confident,” and those in family medicine and internal medicine programs were “mildly confident” (2.3±0.6 and 2.2±0.7, respectively). Even more concerning is the reported low confidence regarding treatment of sight-threatening conditions such as a corneal ulcer and acute angle closure glaucoma. Residents in internal and family medicine programs felt less than “mildly confident” and emergency residents felt “moderately confident” in treating these conditions that require immediate attention. The results of this study are similar to those of a prior Canadian survey in a family medicine residency program that demonstrating residents’ lack of comfort in treating common ophthalmic conditions including corneal erosions and iridocyclitis, as well as sight-threatening conditions including acute angle closure glaucoma and retinal detachment that may result in vision loss if not properly treated in a timely fashion [8].

Among the emergency, internal, and family medicine residents, nearly two-thirds of respondents reported that 1%–5% of their patients presented with ocular complaints. Given these findings, it is imperative that residents be properly educated in assessing patients who present with red eye, decreased vision, or eye pain and when a referral to an specialist is necessary. The ACGME, which sets standards for United States graduate medical education and establishes common requirements for training residents, has no specific requirements regarding how much time is required or dedicated to ophthalmic education in internal, emergency, and family medicine residencies. There is only a mention that internal medicine residents should have the opportunity to experience medical ophthalmology [9]. While recommendations have been made by the Association of University Professors in Ophthalmology to medical school curriculum directors outlining core ophthalmology knowledge [10], we further advocate the inclusion of similar guidelines for internal, emergency, and family medicine residencies.

The major limitation of our study was attempting to reach every resident enrolled in an emergency medicine, internal medicine, and family medicine residency program in the United States at the time of the survey. Contacting residents relied on each program director to forward the survey onto the residents in their program, as well as on the residents completing the surveys. Despite this limitation, we were able to achieve the largest known sample of respondents to evaluate ophthalmology confidence levels and identified a knowledge gap in ophthalmic education and confidence in the management of patients with ocular conditions. We are unaware of any bias, as the survey was sent nationwide and was completely anonymous, except for the potential for respondent bias.

In conclusion, our study demonstrated the current state of ophthalmic training in emergency, internal, and family medicine residencies in the United States. Clinicians in these fields play a crucial role in treating patients with ocular complaints who present to their practice. It should not be assumed that residents have received the necessary ophthalmic education in medical school, as the percentage of required clerkships and didactics is in decline. Inadequate ophthalmic education in primary care residencies may lead to compromised patient care with misdiagnosis of sight-threatening or other ocular conditions, as well as an increased burden on ophthalmologists with non-urgent referrals. Further ophthalmic education is necessary in primary care and emergency medicine residency programs to improve residents’ comfort levels beyond “mildly comfortable” in examining and treating patients and recognizing the need for urgent ophthalmic referrals to improve patient care.

## Figures and Tables

**Table 1.** Descriptive characteristics of all survey respondents in internal, emergency, and family medicine residencies

Characteristic	No. of study population (%)
Total	1,025 (100.0)
Gender	
Male	528 (51.5)
Female	476 (46.5)
Not answered	21 (2.0)
Race/ethnicity	
White	601 (58.6)
Hispanic	72 (7.0)
African-American	26 (2.5)
Asian/Pacific Islander	174 (17.0)
Native American	3 (0.3)
Multiple race/ethnicities	35 (3.4)
Other	74 (7.2)
Not answered	40 (3.9)
Residency program	
Family medicine	271 (26.4)
Internal medicine	412 (40.2)
Emergency medicine	342 (33.4)
Current year	
PGY-1	376 (36.7)
PGY-2	320 (31.2)
PGY-3	291 (28.4)
PGY-4	37 (3.6)
Not answered	1 (0.6)
Medical school	
United States	794 (77.5)
International	227 (22.2)
Not answered	4 (0.4)

PGY, postgraduate year.

**Table 2.** Comparison of ophthalmic education hours and percentage of patients with ocular complaints by family, internal, and emergency medicine residency residents

Characteristic	All respondents	Family medicine	Internal medicine	Emergency medicine	P-value^a)^
No. of respondents	1,025 (100.0)	271 (26.4)	412 (40.2)	342 (33.4)	
Hours in residency dedicated to ophthalmology					<0.001
0–10	809 (79.9)	202 (74.5)	380 (92.5)	227 (66.8)	
11–20	103 (10.0)	17 (6.3)	19 (4.6)	67 (19.7)	
21–30	33 (3.2)	14 (5.2)	2 (0.5)	17 (5.0)	
31–40	27 (2.6)	12 (4.4)	4 (1.0)	11 (3.2)	
>40	50 (4.9)	26 (9.6)	6 (1.5)	18 (5.3)	
Not answered	3 (0.3)				
Patients presenting with ocular complaints (%)					<0.001
<1	273 (26.6)	72 (26.6)	144 (35.0)	57 (16.7)	
1–5	680 (66.3)	178 (65.7)	243 (59.1)	259 (76.0)	
>5	70 (6.8)	21 (7.8)	24 (5.8)	25 (7.3)	
Not answered	2 (0.2)				

Values are presented as number (%).

a) By chi-square test.

**Table 3.** Confidence levels with ophthalmic exams, managing care, and prescribing medications among family, internal, and emergency medicine residents

Survey question	Total	Family medicine	Internal medicine	Emergency medicine
Ophthalmic evaluation				
Visual acuity	3.3±1.2	3.1±1.2	2.9±1.2	3.9±1.0
Pupils	3.8±1.0	3.6±1.0	3.5±1.1	4.3±0.8
Extraocular movements	4.1±0.9	4.1±0.9	3.8±1.0	4.4±0.7
Confrontational visual fields	3.0±1.2	3.0±1.2	2.8±1.2	3.4±1.2
Tonometry	2.1±1.4	1.6±1.0	1.5±1.0	3.3±1.2
Split lamp exam	1.7±1.0	1.6±0.9	1.3±0.7	2.2±1.1
Direct ophthalmoscope	1.9±1.0	2.1±1.0	1.7±0.9	1.8±0.9
Fluorescein dye test	2.6±1.5	2.4±1.3	1.7±1.1	3.9±1.0
External exams				
Blepharitis	2.5±1.2	2.5±1.2	2.2±1.2	2.9±1.2
Chalazion	2.8±1.2	2.9±1.2	2.5±1.2	3.2±1.2
Pre-septal cellulitis	2.8±1.2	2.5±1.1	2.4±1.1	3.4±1.1
Herpes zoster ophthalmicus	2.5±1.2	2.2±1.1	2.2±1.1	3.0±1.2
Managing care				
Conjunctivitis	3.5±1.0	3.6±1.0	3.2±1.1	3.9±0.9
Scleritis	2.3±1.1	2.2±1.1	2.2±1.1	2.6±1.1
Pterygium	2.6±1.4	2.4±1.3	2.3±1.3	3.0±1.4)
Subconjunctival hemorrhage	3.1±1.3	3.0±1.3	2.7±1.3	3.7±1.2
Corneal abrasion	2.8±1.3	2.6±1.1	2.2±1.1	3.8±1.0
Corneal ulcer	2.2±1.2	1.8±1.0	1.7±0.9	3.2±1.2
Herpes keratitis	2.0±1.1	1.7±0.9	2.9±1.1	2.7±1.2
Dry eye	3.1±1.1	3.0±1.1	1.6±0.9	3.3±1.1
Primary open angle glaucoma	1.9±1.0	1.7±0.8	1.6±0.9	2.3±1.0
Angle closure glaucoma	2.0±1.1	1.7±0.9	1.6±0.9	2.8±1.2
Cataract	2.4±1.2	2.2±1.1	2.3±1.2	2.6±1.2
Diabetic retinopathy	2.2±1.1	2.2±1.0	2.4±1.1	2.0±1.0
Hypertensive retinopathy	2.1±1.0	2.0±1.0	2.3±1.1	2.0±1.0
Thyroid eye disease	1.7±1.0	1.6±0.9	1.8±1.0	1.7±1.0
Rheumatologic disease	1.6±0.9	1.5±0.8	1.7±0.9	1.6±0.8
Sjögren syndrome	1.8±0.9	1.8±0.9	1.9±1.0	1.7±0.9
Indications for medication				
Topical antibiotics	2.7±1.1	2.7±1.1	2.2±1.0	3.1±1.1
Topical steroids	2.1±1.0	2.2±1.1	1.9±1.0	2.3±1.0
Topical glaucoma meds	1.9±1.0	1.8±0.9	1.7±0.9	2.3±1.1
Topical nonsteroidal anti-inflammatory drugs	1.8±1.0	1.8±1.0	1.6±0.9	2.1±1.0
Composite score				
Average across all 32	2.5±0.7	2.3±0.6	2.2±0.7	2.9±0.7

Values are presented as mean±standard deviation. Analysis of variance P-values <0.001 across type of residency for each of the 32 ophthalmic measures and the composite score.

**Table 4.** Mean confidence level composite scores by type of residency program (family, internal, or emergency medicine), ophthalmic education hours, and year of training

	Family medicine	Internal medicine	Emergency medicine
Hours in residency dedicated to ophthalmology			
0–10	2.3±0.6	2.2±0.7	2.8±0.7
11–40	2.4±0.6	2.4±0.6	3.1±0.6
>40	3.0±0.8	3.0±0.9	3.3±0.6
ANOVA P-value	<0.001	0.005	<0.001
Current year			
PGY-1	2.2±0.6	2.2±0.7	2.6±0.6
PGY-2	2.3±0.6	2.1±0.6	3.0±0.6
PGY-3	2.5±0.6	2.3±0.7	3.1±0.6
PGY-4	2.9±0.4	2.4±0.7	3.2±0.6
ANOVA P-value	0.008	0.05	<0.001

Values are presented as mean±standard deviation. ANOVA, analysis of variance; PGY, postgraduate year.

## Supplementary materials

Supplement 1. Data files are available from https://doi.org/10.7910/DVN/WBTRMW.

Supplement 2. Audio recording of the abstract.
