# Supplementary material for: Ophthalmology training and competency levels in caring for patients with ophthalmic complaints among United States internal medicine, emergency medicine, and family medicine residents
Source: J Educ Eval Health Prof. 2019 Aug 29;16:25. doi: 10.3352/jeehp.2019.16.25 (PMC6748877; doi:10.3352/jeehp.2019.16.25)
Supplement: Supplementary file 2 [file jeehp-16-25-app.pdf]

Appendix 1. Ophthalmology survey questionnaire to residents developed by Colorado Clinical & Translational Sciences Institute (CCTSI) with the Development and Informatics Service Center (DISC)

CCTSI Colorado Clinical & Translational Sciences Institute

University of Colorado

Colorado Clinical & Translational Sciences Institute (CCTSI) with the Development and Informatics Service Center (DISC)

Ophthalmology Survey to Residents (IM, FM, EM)

Data Dictionary Codebook

07/15/2019 5:53am

#	Variable / Field Name	Field Label<br>*Field Note*	Field Attributes (Field Type, Validation, Choices, Calculations, etc.)
Instrument: **Ophthalmology Needs Assessment Survey** (my_first_instrument)			
1	sid	Record ID	text
2	residency_program	What residency program are you currently enrolled?	radio<br>1 Family Medicine<br>2 Internal Medicine<br>3 Emergency Medicine
3	residency_year	What year are you currently in your residency training?	radio<br>1 PGY 1<br>2 PGY 2<br>3 PGY 3<br>4 PGY 4
4	medschool_where	Where did you complete your medical school?	radio<br>1 United States<br>2 Internationally
5	medschool_hours	How many hours did you have dedicated to Ophthalmology in medical school (through lectures, electives, clerkships)?	radio<br>1 0-10 Hours<br>2 11-20 Hours<br>3 21-30 Hours<br>4 31-40 Hours<br>5 >40 Hours
6	residency_hours	How many hours have you received during your residency training dedicated to Ophthalmology?	radio<br>1 0-10 Hours<br>2 11-20 Hours<br>3 21-30 Hours<br>4 31-40 Hours<br>5 >40 Hours

7	op_training	Have you ever received any Ophthalmology training outside your current curriculum?	radio<br>1 Yes<br>2 No
8	patients_op	What percentage of your patients present with Ophthalmology associated complaints (red eyes, eye pain, blurry vision, etc.)?	radio<br>1 < 1%<br>2 1-5%<br>3 >5%
9	care_va	Section Header: *Please rate your comfort level for managing or coordinating care for the following using the scale: 1 = Not condent 2 = Mildly condent 3 = Moderately condent 4 = condent 5 = = Very condent PERFORMING AN OPHTHALMIC EVALUATION*<br><br>*Visual Acuity*	radio (Matrix)<br>1 Not condent<br>2 Mildly condent<br>3 Moderately condent<br>4 Condent<br>5 Very condent
10	care_pupils	Pupils	radio (Matrix)<br>1 Not condent<br>2 Mildly condent<br>3 Moderately condent<br>4 Condent<br>5 Very condent
11	Extraocular movements	Extraocular movements	radio (Matrix)<br>1 Not condent<br>2 Mildly condent<br>3 Moderately condent<br>4 Condent<br>5 Very condent
12	care_cvf	Confrontational visual	radio (Matrix)<br>1 Not condent<br>2 Mildly condent<br>3 Moderately condent<br>4 Condent<br>5 Very condent
13	care_iop	Tonometry (measure intraocular pressure)	radio (Matrix)<br>1 Not condent<br>2 Mildly condent<br>3 Moderately condent<br>4 Condent<br>5 Very condent
14	care_sle	Split lamp examination	radio (Matrix)<br>1 Not condent<br>2 Mildly condent<br>3 Moderately condent<br>4 Condent<br>5 Very condent

15	care_do	Direct Ophthalmoscope	radio (Matrix)<br>1 Not condent<br>2 Mildly condent<br>3 Moderately condent<br>4 Condent<br>5 Very condent
16	care_fdt	Fluoroscein dye test	radio (Matrix)<br>1 Not condent<br>2 Mildly condent<br>3 Moderately condent<br>4 Condent<br>5 Very condent
17	blepharitis	Section Header: *Please rate your comfort level in coordinating care for: EXTERNAL EXAMS*<br><br>Blepharitis	radio (Matrix)<br>1 Not condent<br>2 Mildly condent<br>3 Moderately condent<br>4 Condent<br>5 Very condent
18	chalazion	Chalazion	radio (Matrix)<br>1 Not condent<br>2 Mildly condent<br>3 Moderately condent<br>4 Condent<br>5 Very condent
19	cellulitis	Pre-septal cellulitis	radio (Matrix)<br>1 Not condent<br>2 Mildly condent<br>3 Moderately condent<br>4 Condent<br>5 Very condent
20	herpes	Herpes zoster opthalmicus	radio (Matrix)<br>1 Not condent<br>2 Mildly condent<br>3 Moderately condent<br>4 Condent<br>5 Very condent
21	conjunctivitis	Section Header: *Please rate your comfort level in managing care for: CONJUNCTIVA and SCLERA*<br><br>Conjunctivitis	radio (Matrix)<br>1 Not condent<br>2 Mildly condent<br>3 Moderately condent<br>4 Condent<br>5 Very condent

22	scleritis	Scleritis	radio (Matrix)<br>1 Not condent<br>2 Mildly condent<br>3 Moderately condent<br>4 Condent<br>5 Very condent
23	pterygium	Pterygium	radio (Matrix)<br>1 Not condent<br>2 Mildly condent<br>3 Moderately condent<br>4 Condent<br>5 Very condent
24	hemorrhage	Subconjunctival hemorrhage	radio (Matrix)<br>1 Not condent<br>2 Mildly condent<br>3 Moderately condent<br>4 Condent<br>5 Very condent
25	abrasion	Section Header: *Please rate your comfort level in managing care for: CORNEA*<br><br>Corneal abrasion	radio (Matrix)<br>1 Not condent<br>2 Mildly condent<br>3 Moderately condent<br>4 Condent<br>5 Very condent
26	ulcer	Corenal ulcer	radio (Matrix)<br>1 Not condent<br>2 Mildly condent<br>3 Moderately condent<br>4 Condent<br>5 Very condent
27	keratitis	Herpes Keratitis	radio (Matrix)<br>1 Not condent<br>2 Mildly condent<br>3 Moderately condent<br>4 Condent<br>5 Very condent
28	dryeye	Dry eye	radio (Matrix)<br>1 Not condent<br>2 Mildly condent<br>3 Moderately condent<br>4 Condent<br>5 Very condent

29	poag	Section Header: *Please rate your comfort level in managing care for: GLAUCOMA and LENS*<br><br>Primary open angle glaucoma	radio (Matrix)<br>1 Not condent<br>2 Mildly condent<br>3 Moderately condent<br>4 Condent<br>5 Very condent
30	acg	Acute ange closure glaucoma	radio (Matrix)<br>1 Not condent<br>2 Mildly condent<br>3 Moderately condent<br>4 Condent<br>5 Very condent
31	cataract	Cataract	radio (Matrix)<br>1 Not condent<br>2 Mildly condent<br>3 Moderately condent<br>4 Condent<br>5 Very condent
32	diabret	Section Header: *Please rate your comfort level in managing care for: OPHTHALMIC COMPLICATIONS OF SYSTEMIC DISEASE*<br><br>Diabetic retinopathy	radio (Matrix)<br>1 Not condent<br>2 Mildly condent<br>3 Moderately condent<br>4 Condent<br>5 Very condent
33	hyperret	Hypertensive retinopathy	radio (Matrix)<br>1 Not condent<br>2 Mildly condent<br>3 Moderately condent<br>4 Condent<br>5 Very condent
34	ted	Thyroid eye disease	radio (Matrix)<br>1 Not condent<br>2 Mildly condent<br>3 Moderately condent<br>4 Condent<br>5 Very condent
35	rheumat	Rheumatological disease (rheumatoid arthritis)	radio (Matrix)<br>1 Not condent<br>2 Mildly condent<br>3 Moderately condent<br>4 Condent<br>5 Very condent

36	sjogrens	Sjogren's	radio (Matrix)<br>1 Not condent<br>2 Mildly condent<br>3 Moderately condent<br>4 Condent<br>5 Very condent
37	meds_antibiotics	Section Header: *Please rate your comfort level in managing care for: INDICATIONS FOR MEDICATION USE*<br><br>Topical antibiotics	radio (Matrix)<br>1 Not condent<br>2 Mildly condent<br>3 Moderately condent<br>4 Condent<br>5 Very condent
38	meds_steroids	Topical steroids	radio (Matrix)<br>1 Not condent<br>2 Mildly condent<br>3 Moderately condent<br>4 Condent<br>5 Very condent
39	meds_glaucoma	Topical glaucoma medications	radio (Matrix)<br>1 Not condent<br>2 Mildly condent<br>3 Moderately condent<br>4 Condent<br>5 Very condent
40	meds_nsaids	Topical NSAIDS	radio (Matrix)<br>1 Not condent<br>2 Mildly condent<br>3 Moderately condent<br>4 Condent<br>5 Very condent
41	suggestions	Thank you so much for participating in this survey, we really appreciate your time!<br><br>This was a pilot survey before it will be sent to many more residents. Please let us know if you have any suggestions.	notes
42	gender	What is your gender?	radio<br>1 Male<br>2 Female

43	race_eth	What is your race/ethnicity?	radio<br>1 White, non-Hispanic<br>2 Hispanic<br>3 African-American<br>4 Asian or Pacific Islander<br>5 Native American<br>6 Multiple race/ethnicities<br>7 Other
44	my_first_instrument_ complete	Section Header: *Form Status*<br><br>Complete?	dropdown<br>0 Incomplete<br>1 Unverified<br>2 Complete
